# Marginal leaf galls on Pliocene leaves from India indicate mutualistic behavior between *Ipomoea* plants and Eriophyidae mites

**DOI:** 10.1038/s41598-023-31393-2

**Published:** 2023-04-07

**Authors:** Taposhi Hazra, Benjamin Adroit, Thomas Denk, Torsten Wappler, Subhankar Kumar Sarkar, Subir Bera, Mahasin Ali Khan

**Affiliations:** 1 Palaeobotany-Palynology Laboratory, Department of Botany, Sidho-Kanho-Birsha University, Ranchi Road, Purulia, 723104 India; 2 Department of Palaeobiology, Swedish Museum of Natural History, Box 50007, 10405 Stockholm, Sweden; 3 IMBE, Aix Marseille Univ, Avignon Univ, CNRS, IRD, Marseille, France; 4 Department of Natural History, Hessisches Landesmuseum Darmstadt, Friedensplatz 1, 64283 Darmstadt, Germany; 5 Paleontology Section, Institute of Geosciences, Rheinische Friedrich-Wilhelms Universität Bonn, 53115 Bonn, Germany; 6 Entomology Laboratory, Department of Zoology, University of Kalyani, Kalyani, Nadia, West Bengal 741235 India; 7 Department of Botany, Centre of Advanced Study, University of Calcutta, 35, B.C. Road, Kolkata, 700019 India

**Keywords:** Ecosystem ecology, Palaeoecology, Herbivory

## Abstract

We report a new type of fossil margin galls arranged in a linear series on dicot leaf impressions from the latest Neogene (Pliocene) sediments of the Chotanagpur Plateau, Jharkhand, eastern India. We collected ca. 1500 impression and compression leaf fossils, of which 1080 samples bear arthropod damage referable to 37 different damage types (DT) in the ‘*Guide* *to* *Insect* *(and* *Other)* *Damage* *Types* *in* *Compressed* *Plant* *Fossils*’. A few leaf samples identified as *Ipomoea* L. (Convolvulaceae) have specific margin galls that do not match any galling DT previously described. This type of galling is characterized by small, linearly arranged, irregular, sessile, sub-globose, solitary, indehiscent, solid pouch-galls with irregular ostioles. The probable damage inducers of the present galling of the foliar margin might be members of Eriophyidae (Acari). The new type of gall suggests that marginal gall-inducing mites on leaves of *Ipomoea* did not change their host preference at the genus level since the Pliocene. The development of marginal leaf galling in *Ipomoea* is linked to extrafloral nectaries that do not offer protection against arthropod galling but indirectly protect the plant against herbivory from large mammals.

## Introduction

Various plant organs including flowers, developing fruits, leaf petioles, and leaves can be exploited by galling arthropods; however, leaves are attacked most commonly^1,2^. Galls may also be induced in mature or already differentiated cells, which have established functions, by means of re-differentiation^3,4^. The most complex gall structures represent a refined tissue organization, whereas simpler galls are typically parenchymatic with poor tissue organization^4^.

Usually, due to their efficient interception of plant photosynthates at the base of the leaf, gall-inducing arthropods achieve higher fitness performance at the proximal compared to the distal portion of the leaf^5,6^. In addition, some galls are preferentially formed at the margins of the leaf because extrafloral nectaries are present in this region of the leaf; a carbohydrate-rich food that attracts some arthropods. These arthropods, in return, may protect the plant by fostering ecologically important protective mutualisms^7^. The feeding behavior of an arthropod is also an important factor in gall development. Sap-sucking insects insert their stylets directly into parenchyma or phloem cells and induce minor modifications to host plant tissues producing simple galls^3,6^. Chewing insects and scrapers-chewers inflict severe damage and consequently induce more complex galls with more specialized tissues^8^.

The occurrence of galls in angiosperms has a diverse and abundant fossil record ^9–14^. Angiosperm radiation during the mid-Cretaceous hosted a major expansion of gall damage types in the early lineages of Austrobaileyales, Laurales, Chloranthales, and Eurosidae and a related increase in arthropod diversity^14^. A few scattered examples of fossil galls in angiosperms are reported from the Cenozoic in India. Important Indian fossiliferous localities depicting records of fossil galls throughout the Cenozoic are of Paleocene^15^, Mio-Pliocene^16,17^, Pliocene^18,19^ and Pleistocene^16^ age. Recently, diverse galls have been reported on fossil angiosperm leaves recovered from the latest Neogene (Pliocene) sediments from the Chotanagpur Plateau, eastern India^20^.

Fossilized galls are typically described and identified based on their size, shape, and sometimes position on the plant organs. On leaves of angiosperms, fossil galls have been described at different positions, such as, including primary, secondary, and tertiary veins, between veins, dispersed throughout the leaf lamina, or on the petiole. All currently known galls within the fossil record are indexed in the *Guide* *to* *Insect* (*and* *Other*) *Damage* *Types* *in* *Compressed* *Plant* *Fossils*^21^.

**Figure 1:**
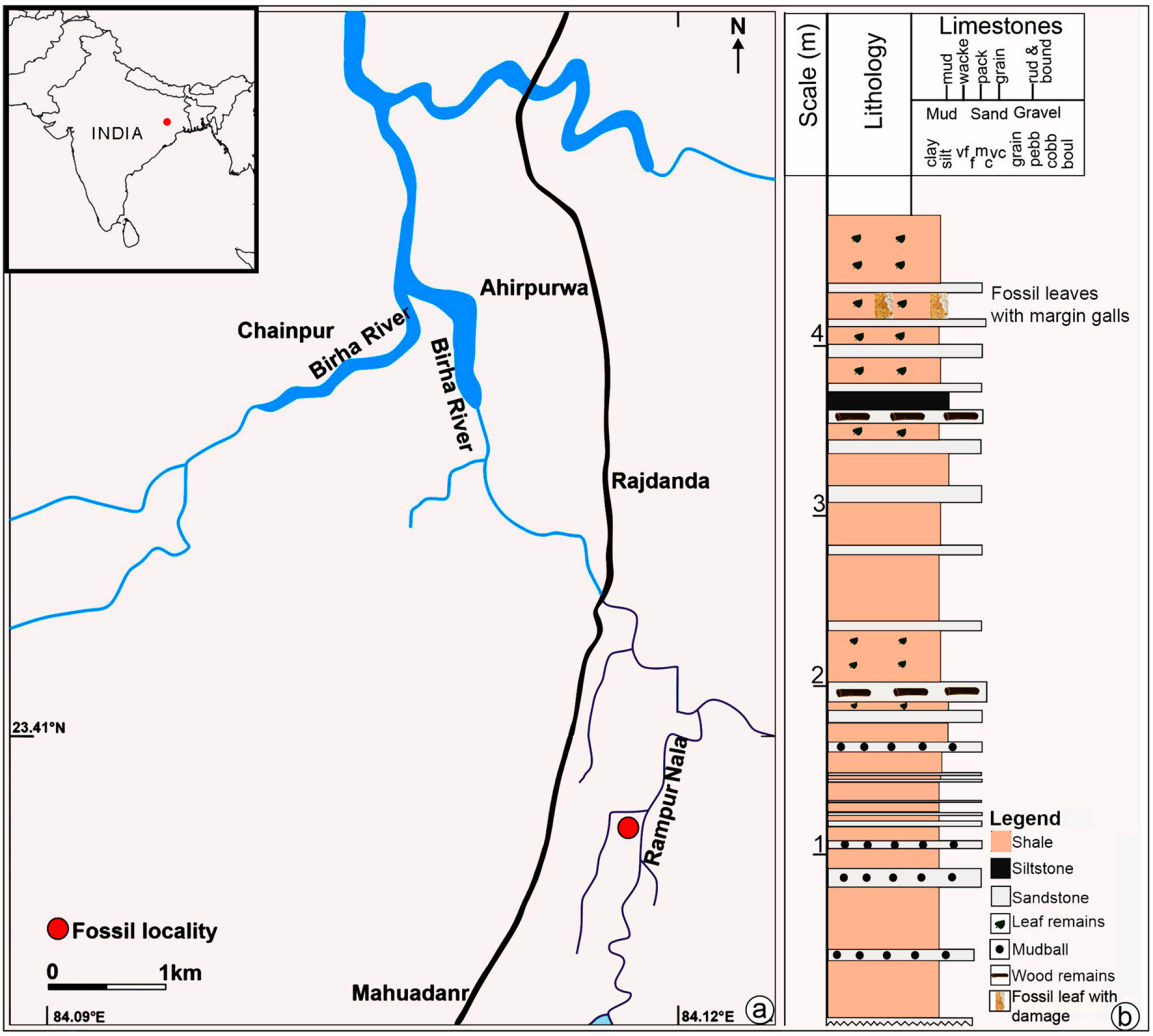
(**a**) Map of India indicating the location of Mahuadanr, Latehar district, Jharkhand (a part of the district resource map of Palamu district, Bihar published under the direction of Director General, Geological Survey of India, Kolkata), along with the fossil locality map; (**b**) generalized lithological section made using SedLog 3.0 software showing litho-units with a composite graphic log of 3 m of exposed sedimentary section.

Here, we document previously unreported well-preserved margin galls arranged in a linear series on dicot leaf impressions from the Pliocene of Chotanagpur Plateau, Jharkhand, eastern India (Fig. 1). We described the damage type (labeled DT413) for inclusion in the guide of plant-arthropod interactions and determined the botanical affinity of the host plant. Further, we explored whether the arthropod-host plant association discovered in latest Neogene strata of India has a counterpart in modern times and how they are geographically connected.

## Results

### Description of the new damage type labeled DT413

**Figure 2:**
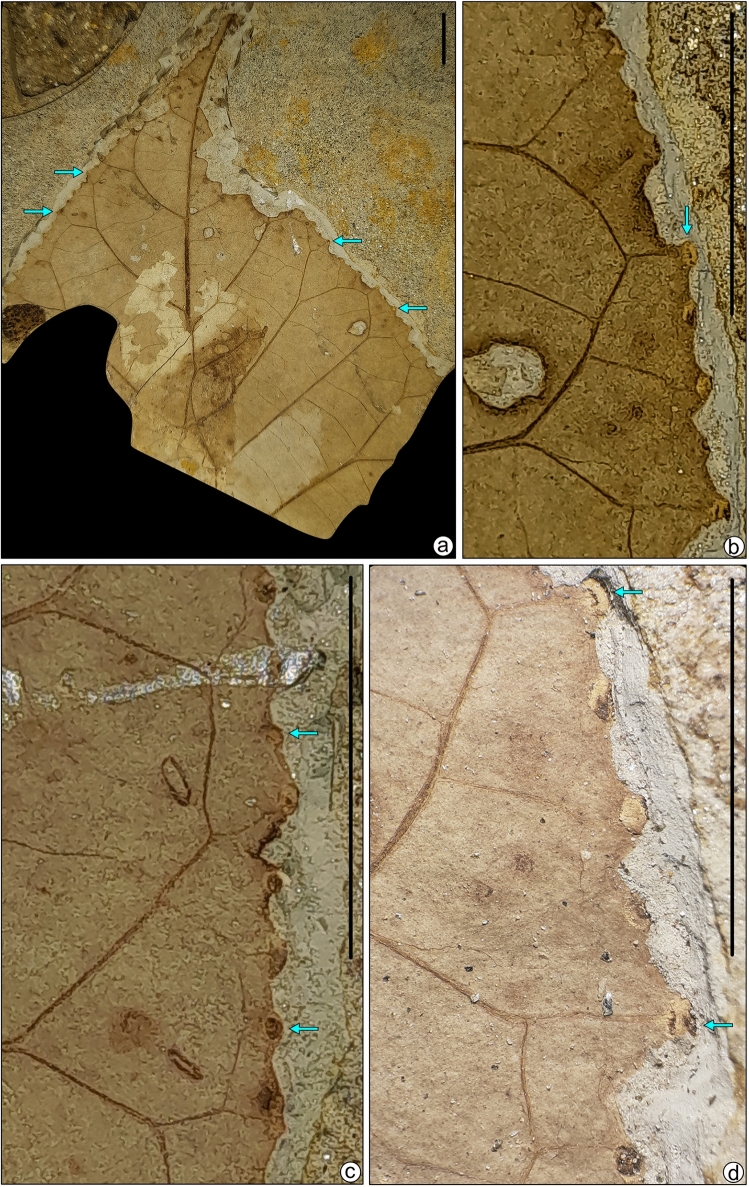
(**a**) Margin gall bearing of fossil leaf samples SKBUH/PPL/JH/325C. (**b–d**) Enlarged view of (**a**); scale bar = 1 cm.

**Figure 3:**
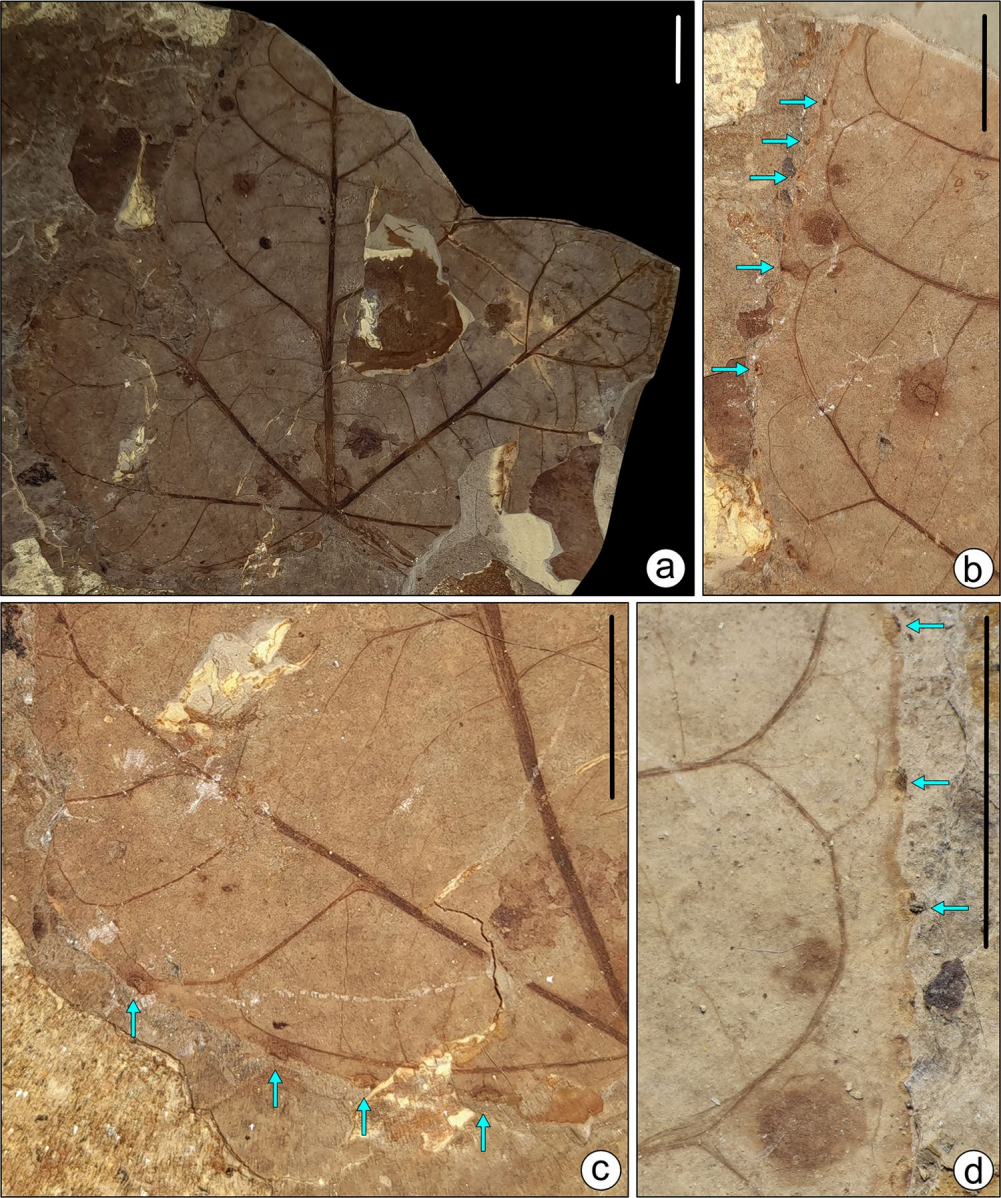
(**a**) Margin gall bearing of fossil leaf specimens SKBUH/PPL/JH/332. (**b–d**) Enlarged view of (**a**); scale bar = 1 cm.

We described angiosperm leaf samples with margin galls in a linear row from the latest Neogene sediments of Jharkhand, eastern India. Galling traces are present along the margin of the lamina in a linear series of small, rounded black dots (Figs. 2, 3, Supplementary Figs. S1, S2; Supplementary Text S1). These series of traces are present in six leaf specimens. We counted tens of galls per leaf damaged by DT413 (Figs. 2, 3; Supplementary Figs. S1, S2). Leaf galls are 0.5–1.5 mm in diameter and 1–4 mm apart, sub-globose or fusiform, solid, hard, and largely hypophyllous, the epiphyllous portion is flat-sausage-shaped, cracked, and fissured, bark-like in general appearance and corky in nature, with slit-like open ostiole in a slight depression and extending the entire length of the gall. The outer surface of the gall is obscurely rugulose and obliquely striate, mostly glabrous. Galls are irregular, solitary, indehiscent, solid, and pouch-galls. They occur as thin emergences on the outer surface of the lamina. The position of the gall is indicated by a small depression, in which the irregular ostiole lies.

### The associated host plant taxa

Clade: Asterids

Order: Solanales Juss. ex Bercht. & J.Presl

Family: Convolvulaceae Juss., nom. cons.

Genus: cf. *Ipomoea* L. (Supplementary Figs. 2a, 3a, S1a, S2a, S3a,b)

Leaves simple, symmetrical; lamina unlobed to lobed, two leaf samples trilobed, four specimens unlobed, lamina mesophyllous, preserved length of lamina ~ 6 to 10 cm, width 8‒12 cm; lamina shape ovate to broadly ovate; base cordate to sub-cordate; apex acute to acuminate, acuminate apex well preserved in one specimen, acumen ~ 2 cm long; lamina margin entire; petiole well preserved in one specimen, long, prominent, ~ 3 cm in length; primary venation suprabasal actinodromous with five to seven primary veins diverging radially from a single point; secondary venation continuous brochidodromous, festooned type, secondary veins sharply curved near the margin, irregularly spaced, their angle of divergence acute; tertiary venation percurrent, oblique, predominantly alternate and distant; quaternary veins faint, reticulate.

Remarks: We investigated well-preserved macromorphological features of leaf impressions and compared our fossil specimens with extant angiosperm leaves having a broad ovate heart-shaped lamina with a cordate base, entire margin, actinodromous primary venation, and brochidodromous secondary venation (Supplementary Table S1). On critical examination, we found that the Pliocene fossil specimens are quite similar to modern leaves of six taxa, namely, *Cercis* *canadensis* L. (Fabaceae), *Tinospora* *cordifolia* (Willd.) Hook. f. and Thomson (Menispermaceae), *Matelea* *carolinensis* (Jacq.) Woodson (Apocynaceae), *Catalpa* *bignonioides* Walter (Bignoniaceae), *Ipomoea* sp. (Convolvulaceae), and *Thespesia* *populnea* (L.) Sol. ex Corrêa (Malvaceae) (Supplementary Table S1). Modern leaves of *C.* *bignonioides* and *T.* *populnea* differ in the macrophyllous type of lamina with a long petiole (˃ 70 mm). On the contrary, our fossil specimens possess a mesophyllous type of lamina and a short petiole (≤ 60 mm). *M.* *carolinensis* differs in having opposite percurrent tertiary veins, while our specimens possess alternate percurrent tertiary veins. *Cercis* *canadensis* differs in having 7–9 main veins, while our specimens exhibit 3–5 main primary veins. In *T.* *cordifolia,* the costal secondary veins are opposite. In contrast, the costal secondary veins of our fossil specimens are alternate. Therefore, the size, shape (ovate), apex (acuminate), base (cordate), suprabasal actinodromous primary venation, festooned brochidodromous secondary veins, and percurrent tertiary veins of the recovered fossil leaf samples show the closest resemblance to modern leaves of *Ipomoea* of the family Convolvulaceae*.*

### Overview of damages to leaf assemblage

Of the 1500 fossil angiosperm leaves studied, 1080 leaves (72%) were damaged. Approximately 37 damage types (DT), representing six functional feeding groups (FFGs; see Material & Methods section), were identified based on the *Guide* *to* *insect* *damage* *types*. Galling constituted 50.74% of all damage-type occurrences, followed by margin feeding (23.24%), hole feeding (17.04%), surface feeding (3.33%), leaf-mining (2.22%), and skeletonization (1.94%).

## Discussion

Our comparison of DT413 to analogous modern galls was based on: (i) gall features observed in modern galls; (ii) the likelihood of a particular galling culprit lineage being present considering phylogenetic evidence; and (iii) modern arthropod species causing margin galls occurring in the same plant taxon today. Unlike modern galls, the recognition of fossil galls may be complicated due to the varying state of their preservation. Galls induced by arthropods (mainly insects) on leaves are diverse in the fossil record^11,14,20–24^. The identification of fossil galls is usually based on gross morphology and position. Teams of researchers used different morphological criteria (size, shape, leaf position, nature of the gall wall, nature of the exit pore, and comparison with recent specimens) to describe and categorize fossil galls^10^. Leaf galls have previously been observed on veins (either the primary, secondary, or tertiary veins), between veins, near the lamina apex or base, and dispersed throughout the lamina and petiole. In fact, to the best of our knowledge, none of the fossil galls described in previous studies resemble the galls on the leaf margin presented here.

Labandeira et al. (2007)^21^ and extended unpublished updates which are used as the main reference in current studies of plant-arthropod interactions in the fossil record, described and classified all known fossil types of galls based on a large worldwide dataset. In addition to gross morphological characteristics and whether galls are solitary or in groups, they also recognized different types of galls based on their position on the leaves (Supplementary Fig. S4). For example, circular to elliptic fossil galls avoiding major veins (DT 32), circular to elliptic galls on primaries (DT 33), circular to elliptic galls on secondary veins (DT 34), galls on the petiole (DT 55). The marginal gall traces reported here do not match any galling DT previously described and are therefore proposed as a new DT. Fossil leaf remains of the genus *Ipomoea* from Pliocene strata of the Jharkhand region (eastern India) currently are their only known host specimen. Our description follows the style of the *Guide* *to* *insect* *damage* *types*^21^ and is here designated as DT 413 (Supplementary Text S1).

It is difficult and sometimes impossible to figure out which arthropod taxa may have caused damage to fossil foliage because a single arthropod species may produce multiple types of damage while at the same time, certain damages can be made by many different arthropod species^25^. Nevertheless, galling represents such a specific interaction between a plant and an arthropod species that it is possible to presuppose which arthropod taxon could have caused it in the fossil record. Overall, gall producers mostly belong to the insect orders Hemiptera, Diptera, Hymenoptera, and within Arachnida the 'superorder' Acariformes^26^. Among these arthropod taxa, the present marginal fossil galls lie well within the limits of size, shape, and other morphological characteristics exhibited by present-day Eriophyidae (Acariformes) mite-producing galls that occur mainly on plants belonging to the family Rosaceae and Convolvulaceae^27,28^. The fossil galls share relevant characteristics (size, shape, position on the leaf blade, nature of gall wall, and nature of ostiole or exit pore of the gall) with marginal galls produced by the acarian genus *Eriophyes* on the plant genus *Prunus* (Rosaceae)^29^. Based on this morphological similarity, we could suggest that damage inducers of our marginal galls might have been closely related to the gall mite *Eriyophes*. The *Eriophyes* galls on *Ipomoea* species have been reported primarily on stems and rarely on leaves ^28^. However, there is another species of Eriophyidae (*Aceria* *gastrotrichus*) that has been observed on *Ipomoea* *staphylina*^30^ causing a similar type of margin galls, and these galls are native to India^31,32^. This would appear to support our initial taxonomic determination of the host plant as belonging to the genus *Ipomoea* and highlights how a combination of morphological and physiological traits can contribute to a more reliable determination and understanding of fossil plant taxa.

These gall mites feed by puncturing plant cells with stylets and sucking the cell contents. They frequently infest buds and young leaves that cause damage and are easily overlooked^33^. They are quite host-specific, monophagous, usually confined to one plant genus, or, at most, members of a single family, and phytophagous mites cause direct damage to plants by sucking plant sap and forming intimate relationships with their host plants that are similar to the one described in the present study^34,35^.

The fossil leaf specimens, on which the marginal galls are situated, are not completely preserved. By combining all the characteristics of the different leaf samples and comparison with abundant extant leaf material, we suggested that the host plant for the newly discovered galling type might belong to the genus *Ipomoea* (Convolvulaceae). This taxon, commonly known as ‘morning glory’, is a pantropical genus that grows naturally in warm temperate and subtropical regions of the world^36,37^. Traditionally an American origin was suggested for *Ipomoea* based on the most ancient fossil record^38,39^, or in East Gondwana based on molecular data^40^. Recent paleobotanical studies tend to support an East Gondwana origin in the Paleocene^41^ when India still was part of Gondwana.

To narrow down our identification below the genus level, we consulted the catalogs of the Global Biodiversity Information Facility (https://www.gbif.org) and the Indian Virtual Herbarium (https://ivh.bsi.gov.in) of *Ipomoea* species. Among the numerous species examined*,* the unlobed and trilobed leaves of *I.* *purpurea* (L.) Roth provide the closest match with our fossil specimens in terms of leaf organization (shape, primary, and complex higher-order venation Supplementary Table S1; Supplementary Fig. S3) and thus can serve as the “modern analogue” of the fossil specimens.

However, the widespread common species *I.* *purpurea* is thought to have originated in tropical America^42^ and only in historical times has been introduced in eastern Asian countries^43^.

Importantly, however, many *Ipomoea* species are woody climbers, and this habit may constrain leaf morphology and variability (frequent change from unlobed to lobed leaves in individual plants) as is the case in other climbers (cf. *Aristolochia* L., *Clematis* L.*,* *Hedera* L., other Convolvulaceae, among many others)^44–46^. Therefore, one might expect a high degree of convergent leaf evolution in the large genus *Ipomoea*. Highly similar leaf morphologies may be present in distantly related species, whereas markedly different leaf morphologies may occur in closely related species. Thus, we do not infer closer taxonomic (and biogeographic, phylogenetic) relationships of our fossil leaves with a particular modern species.

Other than morphological traits, a great number of physiological traits can be used to circumscribe modern plant taxa. Among these, the plant defense strategy of a particular plant may provide important ecological and taxonomic (phylogenetic) information^47^.

With this in mind, we have extensively surveyed modern tropical forests adjacent to the fossil locality to compare our fossil leaf specimens and their unique galls with modern angiosperm leaves that bear galls. So far, we have not noticed any marginal galls on modern leaves in nearby modern environments. Reviewing the 'Plant Galls of India' by Mani (1973)^48^, it turned out that similar types of feeding traces (i.e., margin galls) do occur on modern leaves of different angiosperm taxa such as *Terminalia* *arjuna* (Roxb.) Wight and Arn. (Combretaceae), *Madhuca* *longifolia* (L.) J.F. Macbr. (Sapotaceae), *Avicennia* *officinalis* L. (Avicenniaceae), *Piper* *nigrum* L. (Piperaceae), *Loranthus* Jacq. (Loranthaceae), *Ficus* *drupacea* Thunb. (Moraceae), *Schima* *wallichii* (DC.) Korth. (Theaceae), *Caryocar* *brasiliense* Cambess. (Caryocaraceae) and *Ipomoea* *staphylina* Roem. and Schult. (Convolvulaceae) (Table 1; Supplementary Table S2; Supplementary Fig. S5). More detailed surveys of *Ipomoea* species in their natural environment are needed to fully document their gall diversity.

**Table 1:** Comparative morphological chart of modern margin galls of various angiosperm leaves related to fossil margin galls of our recovered fossil leaves similar to *Ipomea* sp. More details in Supplementary Table S2.

Plant hosts	Causal organisms	Gall shape	Gall diameter	Ostiole	Reference
*Terminalia* *arjuna* (Combretaceae)	*Megatrioza* *hirsutam* (Homoptera)	Subspherical	2 mm	Present	Mani, 1973^48^
*Madhuca* *longifolia* (Sapotaceae)	Itonididae (Diptera)	Hemispherical or strongly convex	1–5 mm	Absent	Mani, 1973^48^
*Avicennia* *officinalls* (Acanthaceae)	*Eriophyes* sp. (Acarina)	Sub-globose	2–4	Present	Mani, 1973^48^
*Loranthus* sp. (Loranthaceae)	Psyllidae (Homoptera)	Ovoid, ellipsoid or sub-globose	10–15 mm long and 5–8 mm thick	Absent	Mani, 1973^48^
*Ficus* *drupacea* (Moraceae)	Orthoptera (?)	Subglobose or fusiform	10–15 mm	Absent	Mani, 1973^48^
	*Pauropsylla* *globuli* (Homoptera)	Globose	5–6 mm		Mani, 1973^48^
*Caryocar* *brasiliense* (Caryocaraceae)	*Eurytoma* sp. (Hymenoptera)	Spherical	4–6 mm	Present	Cintra et al., 2020^63^
*Quercus* sp.	*Andrlcus* *pruinosus*, *A.* *utriculus*, *A.* *pilulus*; *Neuroterus* *bassettii*; *N.* *cockerell*i; *Dryophanta* *pulchripennis*	Globose	2–3 mm	Present	Felt, 1940^27^
*Ipomoea* *staphylina* (Convolvulaceae)	*Eriophyes* sp. (Acarina)	Globose	2–3 mm	Present	Amante et al., 2003^28^
*Schima* *wallichii* (Theaceae)	*Trioza* sp. (Homoptera)	Elongate	10–12 mm long and 4–7 mm thick	Present	Mani, 1973^48^
*Alstonia* *scholaris* (Apocynaceae)	*Pauropsylla* *tuberculata* (Psyllidae)	Globose	3–6 mm	Present	Our field survey
Fossil margin galls on *Ipomoea-*like fossil leaves	Eriophyid mites	Globose	0.5–1.5 mm	Present	This paper

The role of marginal galls on angiosperm leaves is not completely understood. Different plant taxa have extrafloral nectaries (EFNs) along the leaf margin, such as *Saraca* sp., *Aporusa* sp.^49^, *Prockia* sp.^50^, *Caryocar* sp.^51^ and many others. EFNs are notable, as they are used to attract some small arthropods, which come for nutrients, and in turn, protect the leaves (and other plant organs) from more harmful herbivores as a mutualistic interaction^7,52^. The presence of EFNs at leaf margins could influence arthropod-galling preference^51^. Among the tens of plant families that bear EFN^53^ are also species of *Ipomoea*^52,54^. Different studies have been conducted on the role of EFNs in the petiole, pedicel, and leaves of the American species of *Ipomoea*^52,55^. These studies found a clear defense function in nectaries on sepals in species of *Ipomoea* often in combination with chemical antiherbivore components in leaf tissues (alkaloids^56^), but even if *Ipomoea* leaves can also include EFNs^57^ their functions remain to be fully understood^58^.

The marginal galling observed in the Pliocene and extant species of *Ipomoea* from eastern India associated with leaf margin nectaries provides evidence for extrafloral nectaries that do not have a defense function against galling arthropods but possibly prevented these leaves from herbivory. A number of have shown that the presence of galls had a repelling effect against mammal herbivory^59^ or other arthropod herbivory^60^ from leaf consumption.

## Material & methods

During paleobotanical fieldwork conducted between 2018 and 2022, a large number of compressed and impressed fossil leaves including margin galls were recovered from river-cutting sections of the latest Neogene (Pliocene) sediments of Mahuadanr Valley (23.3965°N, 84.1066°E; altitude 353 m a.s.l.), Jharkhand, Chotanagpur Plateau, eastern India (Fig. 1). The sedimentary rock section is exposed over a length of approximately 100 m and extends to a maximum thickness of 5 m on the left bank of Rampur Nala. The lithology in the studied section includes mainly shales and sandstone. A detailed description of the section yielding the plant fossils studied for the present study has been provided in previous studies^20,61^. A Pliocene age has been suggested for the studied sedimentary strata based on lithostratigraphic correlation and plant macrofossils^61,62^.

We have collected 1500 fossil leaves from Pliocene sediments, of which 1080 leaf samples bear arthropod damage types belonging to six functional feeding groups (FFGs): Hole feeding, margin feeding, surface feeding, mining, galling, and skeletonization. We follow the *Guide* *to* *insect* *damage* *types* for the identification of damage patterns. Galling is the prevalent form of damage here. Various types of galls were observed on the primary, secondary, and tertiary veins of fossil leaves. Interestingly, among these leaves, we noticed six fossil leaf specimens bearing galls on the leaf margin in a linear series, which is unique.

**Figure 4:**
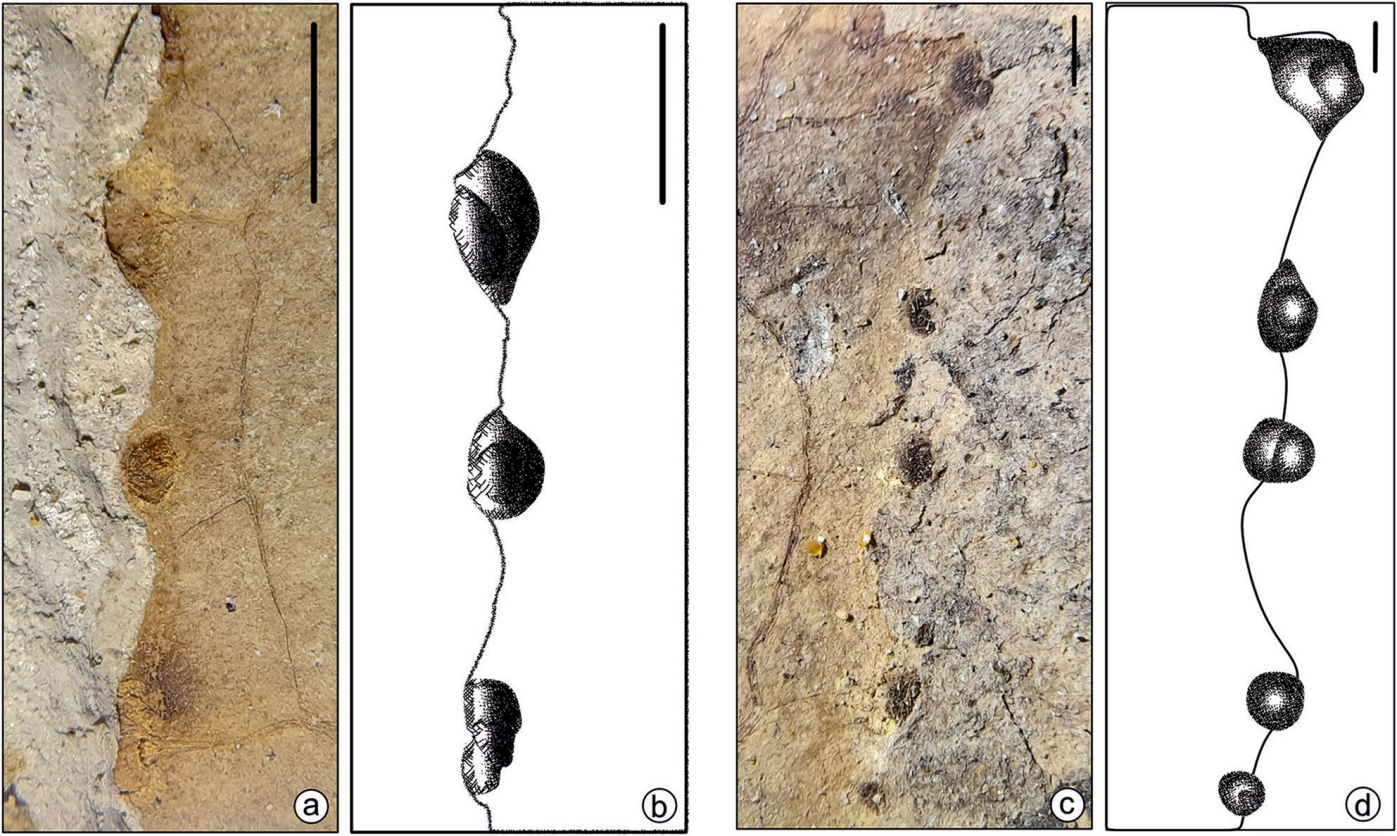
Enlargement and line drawings of marginal galls. Specimen SKBUH/PPL/JH/325C (**a,b**); specimen SKBUH/PPL/JH/324A (**c,d**). Scale bar = 1 mm.

Fossil specimens bearing marginal galls (Figs. 2, 3 and 4; Supplementary Figs. S1, S2, S6) required preparation before photography because the rather small galls were not well-exposed in the initial fracture. Therefore, the overlying matrix was removed with fine needles, scalpels, and brushes. After cleaning, photographs were taken using a digital camera (Canon EOS 1500D) mounted on a stereo zoom microscope, and edited with CorelDraw and Adobe Photoshop software (Figs. 2, 3). The details of the margin galls were drawn using CorelDraw ver. 2021 (Fig. 4; Supplementary Fig. S6, S7).

We also surveyed modern-day forests adjacent to the fossil exposures for similar types of galling on modern leaves and photographed and collected diverse gall-bearing modern leaves. The terminology adopted by Mani (1973)^48^ was followed for the general description of the margin galls. The fossil specimens (SKBUH/PPL/JH/324A, SKBUH/PPL/JH/324B, SKBUH/PPL/JH/325A, SKBUH/PPL/JH/325B, SKBUH/PPL/JH/325C, SKBUH/PPL/JH/332) reported here are housed at the Department of Botany, Palaeobotany and Palynology Laboratory, Sidho-Kanho-Birsha University, Purulia, India. All the methods of modern leaf sampling and plant studies were carried out following the “IUCN Policy Statement on Research Involving Species at Risk of Extinction”.

## Supplementary Information

Supplementary Legends.

Supplementary Figure S1.

Supplementary Figure S2.

Supplementary Figure S3.

Supplementary Figure S4.

Supplementary Figure S5.

Supplementary Figure S6.

Supplementary Figure S7.

Supplementary Table S1.

Supplementary Table S2.

Supplementary Information.

## Data Availability

All data from this study are included in the present publication and its Supplementary Material.
