# Supplementary material for: Marginal leaf galls on Pliocene leaves from India indicate mutualistic behavior between Ipomoea plants and Eriophyidae mites
Source: Sci Rep. 2023 Apr 7;13:5702. doi: 10.1038/s41598-023-31393-2 (PMC10082081; doi:10.1038/s41598-023-31393-2)
Supplement: Supplementary file 1 — Supplementary Legends. [file 41598_2023_31393_MOESM1_ESM.docx]

**Captions of Supplementary materials**

**Supplementary Figure S1.** Marginal galls on latest Neogene (Pliocene) leaves of Mahuadanr. (a-b) fossil leaf of marginal galls bearing (a) specimen no. SKBUH/PPL/JH/324A; (b) specimen no. SKBUH/PPL/JH/324B). (c) enlarged view of (a). (d) enlargement of (b); Scale bar = 1 cm.

**Supplementary Figure S2.** Marginal gall on the fossil leaf specimen (a) SKBUH/PPL/JH/325A and (b) SKBUH/PPL/JH/325B; (c) enlarged view of (a). (d) enlargement of (b); Scale bar = 1 cm.

**Supplementary Figure S3.** Fossil and the modern leaf of *Ipomoea*. (a) fossil leaf of *Ipomea* (specimen no: SKBUH/PPL/JH/324A) showing a broadly ovate-shaped lamina, a cordate base, and an actinodromous venation pattern. (b) modern leaf of *Ipomoea* showing similar nature of the apex, base, shape, size, and primary venation pattern. (c) enlargement of the fossil specimen showing the venation pattern (lateral primary vein marked by a blue arrow; tertiary veins marked by a white arrow; marginal loop marked by a yellow arrow). (d) enlargement of the modern leaf of *Ipomoea* showing the venation pattern; (lateral primary vein marked by a blue arrow; tertiary veins marked by a white arrow; marginal loop marked by a yellow arrow); Scale bar = 1 cm.

**Supplementary Figure S4**. Schematic representation of various types of foliar galls based on their position reported by Labandeira et al., (2007)^21^ and the present foliar marginal gall type. (a) circular to ellipsoidal; avoid major veins (DT 32). (b) circular to ellipsoidal; in primaries (DT 33). (c) circular to ellipsoidal; on 2° veins, 0.5 - 1.1 mm wide (DT 34). (d) on the petiole (DT 55). (e) small, hemispherical; thoroughly carbonized; diameters ~ 0.1 - 1.0 mm; 1° and 2° veins avoided (DT 80). (f) elongate, elliptical, striated; centered on major veins, especially a midvein (DT 85). (g) large; on 3° veins, ovoidal-circular; central chamber sharply separated from thick carbonized brim (DT 110). (h) our recovered fossil galls on the leaf margin (DT413, presented in this manuscript)

**Supplementary Figure S5.** (a) Galls on a leaf of *Ipomoea staphylina*, photo modified from Amante et al., (2003)^28^. (b) Galls on a leaf of *Alstonia scholaris*. (c) Galls of *Eurytoma* sp. (Hymenoptera: Eurytomidae) on leaf margin of *Caryocar brasiliense*., photo modified from Cintra et al. (2020)^63^. (d) Line drawing of foliar marginal galls on a leaf of *Ficus drupacea* (no. 715) modified after Mani (1973)^48^.

**Supplementary Figure S6.** Enlargement and line drawings of marginal galls. SKBUH/PPL/JH/324A (a–b); SKBUH/PPL/JH/325A (c–d); SKBUH/PPL/JH/324A (e–f); SKBUH/PPL/JH/325C (g–h); SKBUH/PPL/JH/332 (i–l). Scale bar = 1 mm.

**Supplementary Figure S7**. (a-d), Line drawing of fossil specimens showing marginal galls (a) SKBUH/PPL/JH/324A; (b) SKBUH/PPL/JH/332; (c) SKBUH/PPL/JH/325C; (d) SKBUH/PPL/JH/325A). Scale bar = 1 cm.
