# Supplementary figures and images for: Marginal leaf galls on Pliocene leaves from India indicate mutualistic behavior between Ipomoea plants and Eriophyidae mites
Source: Sci Rep. 2023 Apr 7;13:5702. doi: 10.1038/s41598-023-31393-2 (PMC10082081; doi:10.1038/s41598-023-31393-2)

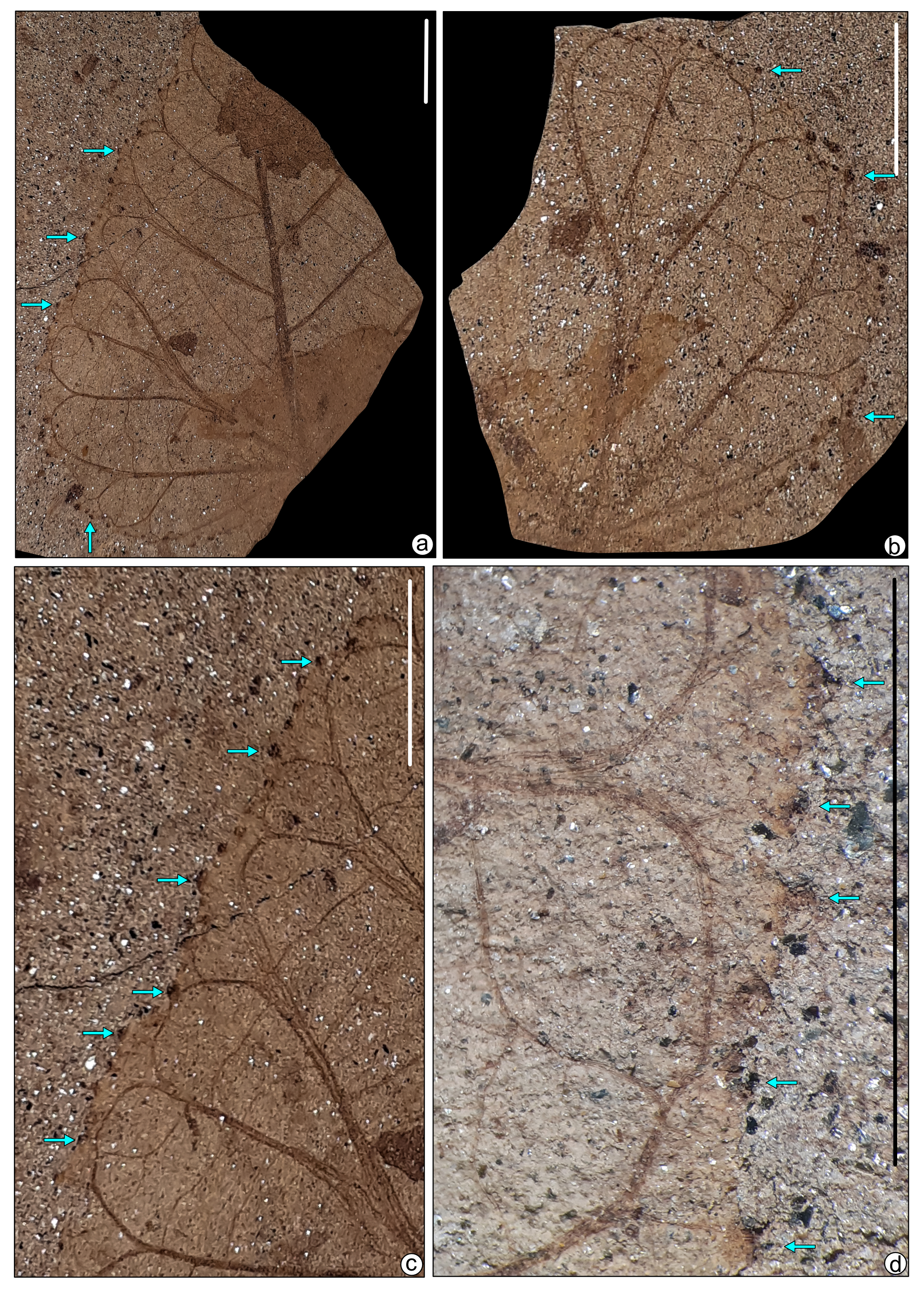

Supplement: Supplementary file 2 — Supplementary Figure S1. [file 41598_2023_31393_MOESM2_ESM.jpg]

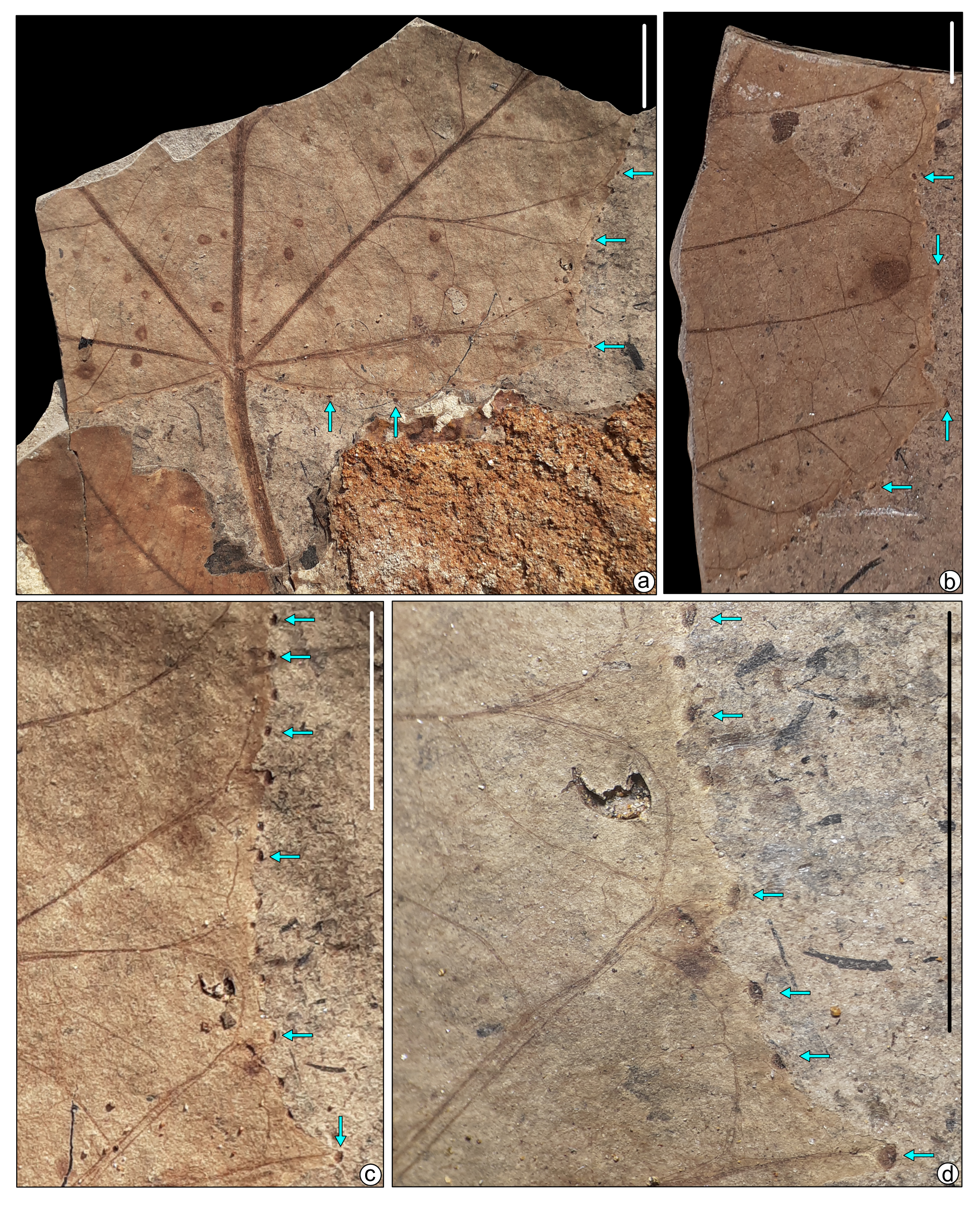

Supplement: Supplementary file 3 — Supplementary Figure S2. [file 41598_2023_31393_MOESM3_ESM.jpg]

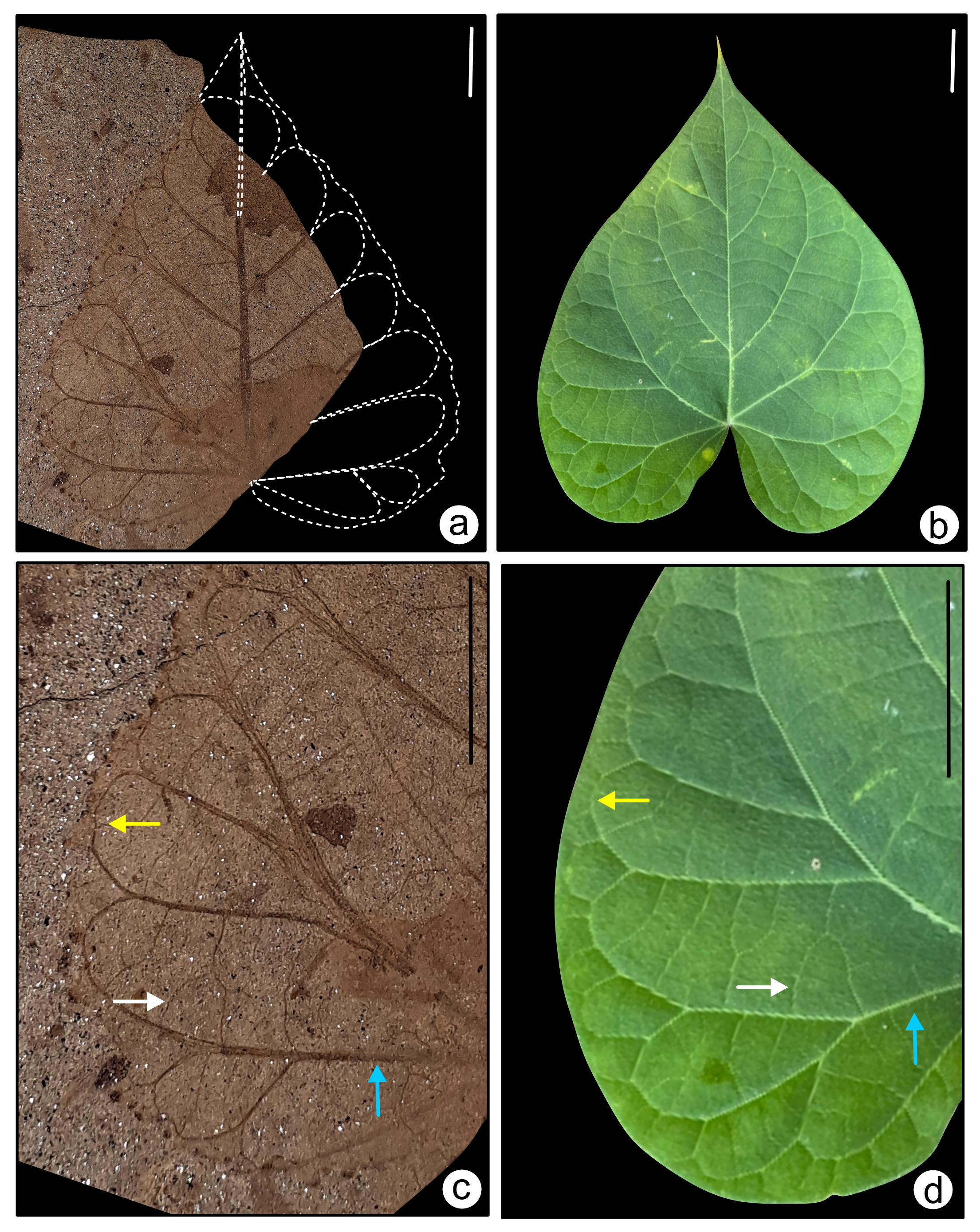

Supplement: Supplementary file 4 — Supplementary Figure S3. [file 41598_2023_31393_MOESM4_ESM.jpg]

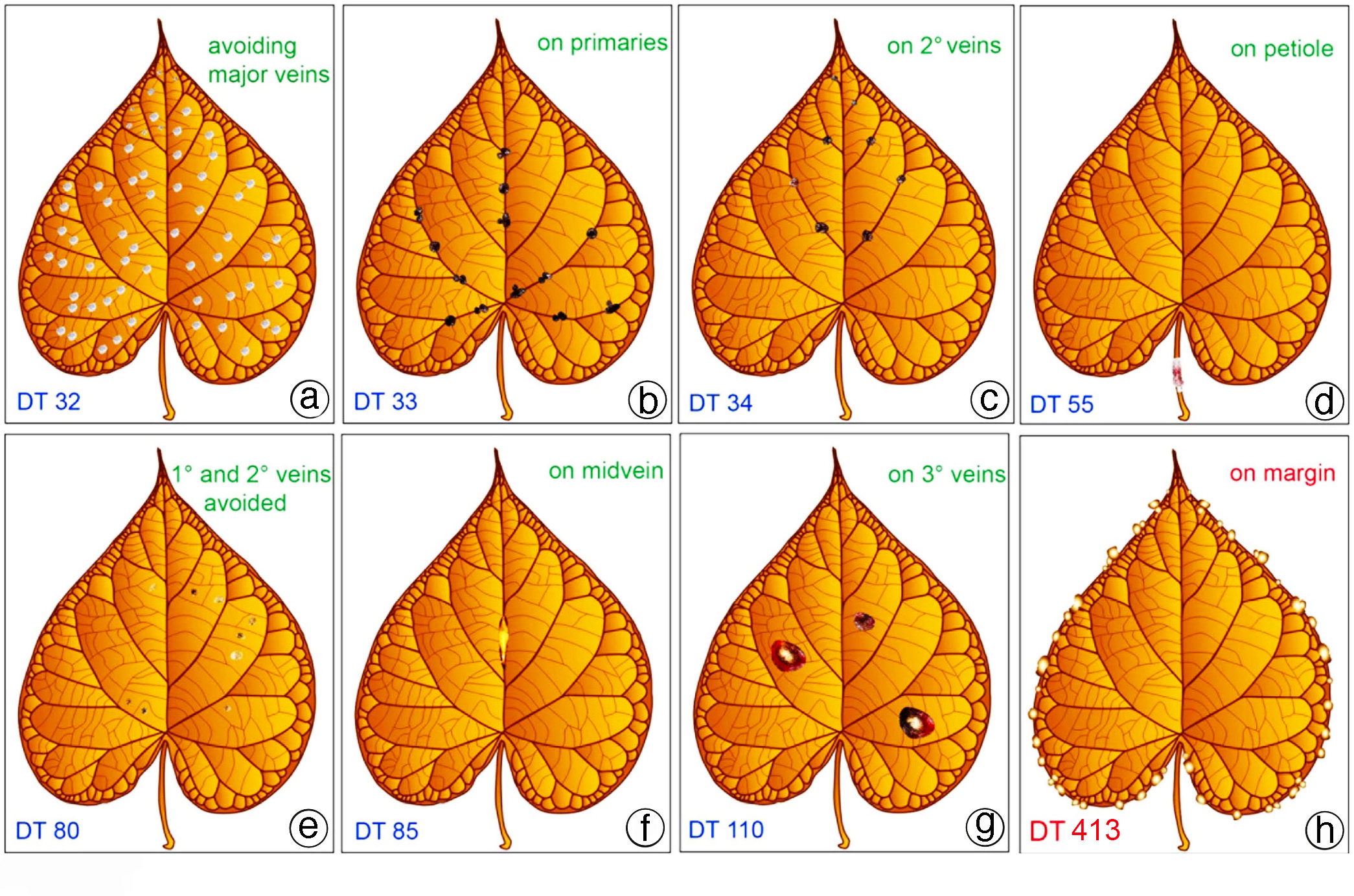

Supplement: Supplementary file 5 — Supplementary Figure S4. [file 41598_2023_31393_MOESM5_ESM.jpg]

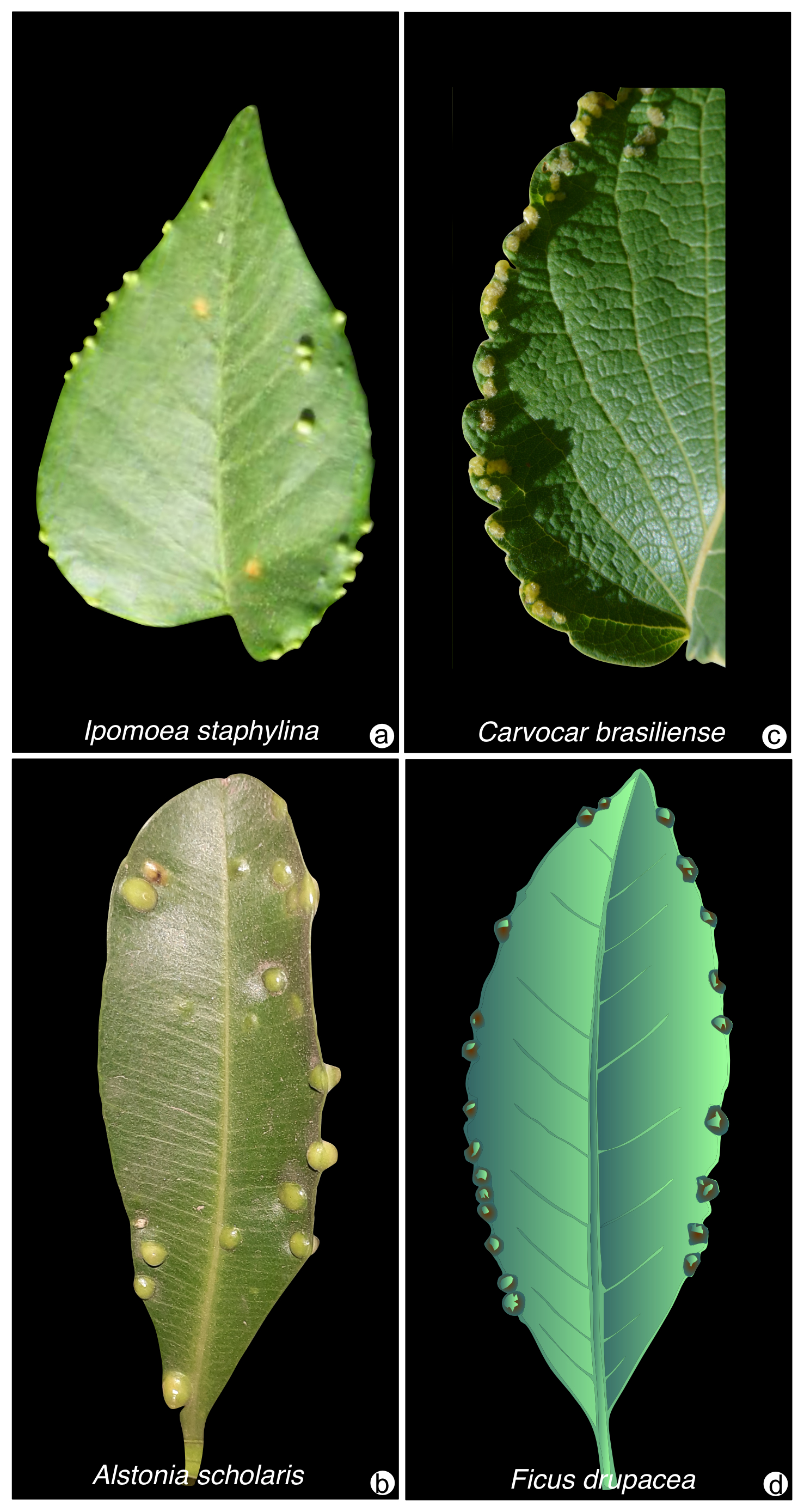

Supplement: Supplementary file 6 — Supplementary Figure S5. [file 41598_2023_31393_MOESM6_ESM.jpg]

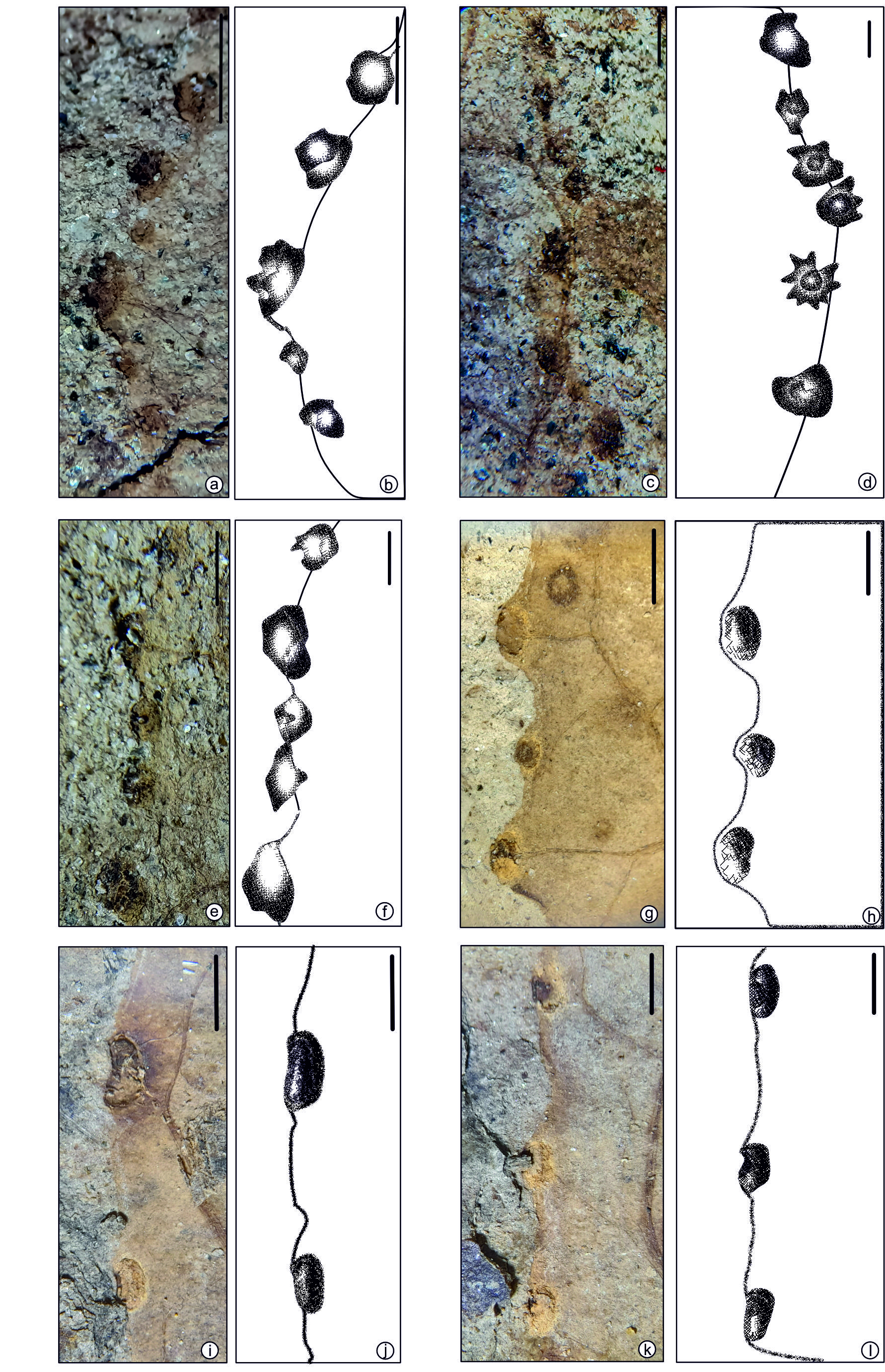

Supplement: Supplementary file 7 — Supplementary Figure S6. [file 41598_2023_31393_MOESM7_ESM.jpg]

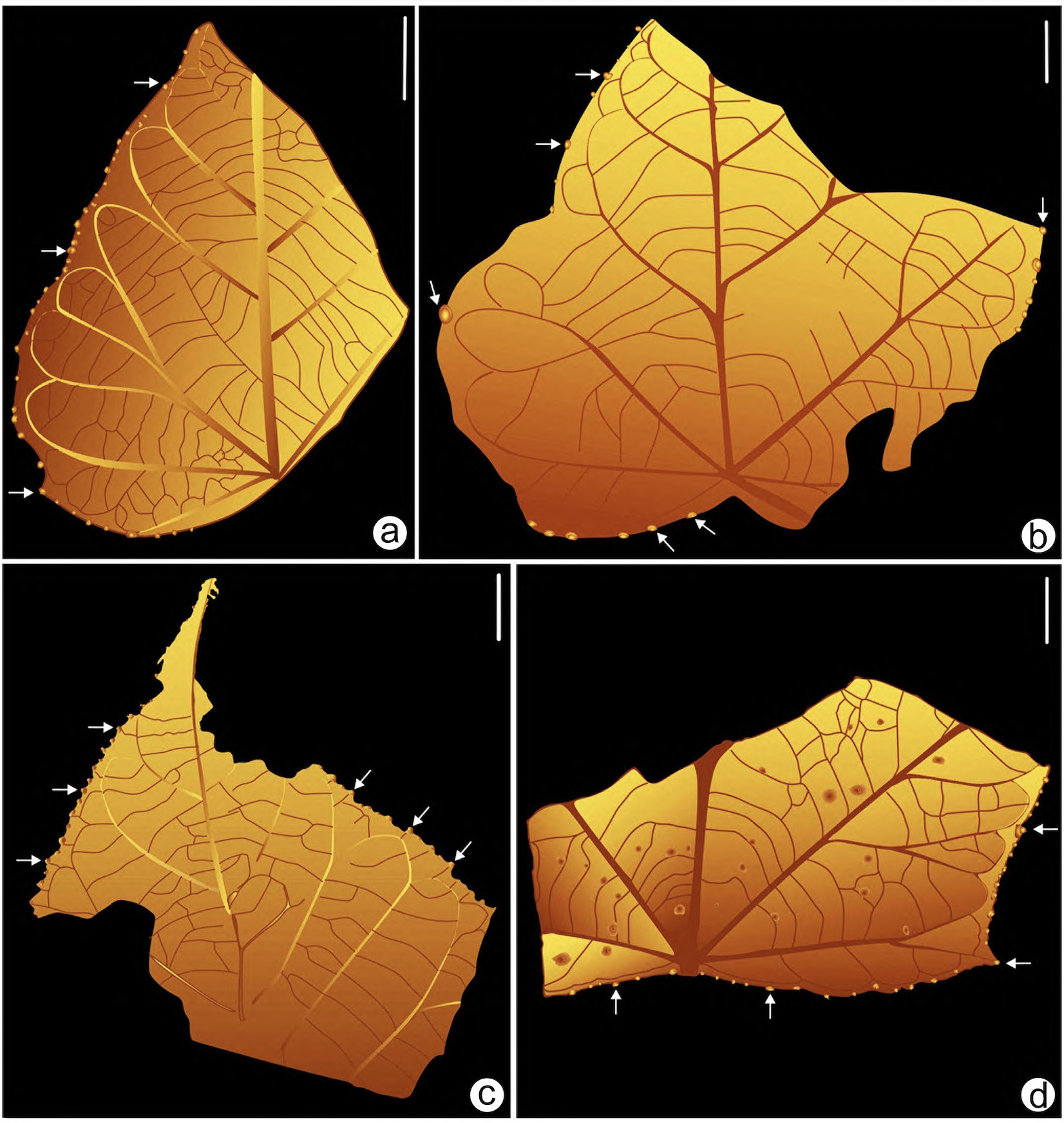

Supplement: Supplementary file 8 — Supplementary Figure S7. [file 41598_2023_31393_MOESM8_ESM.jpg]
