# Supplementary material for: Marginal leaf galls on Pliocene leaves from India indicate mutualistic behavior between Ipomoea plants and Eriophyidae mites
Source: Sci Rep. 2023 Apr 7;13:5702. doi: 10.1038/s41598-023-31393-2 (PMC10082081; doi:10.1038/s41598-023-31393-2)
Supplement: Supplementary file 9 — Supplementary Table S1. [file 41598_2023_31393_MOESM9_ESM.doc]

**Table S1: List of plant species morphologically related to the taxa *Ipomea***

| **Species** | **Apex** | **Base** | **Margin** | **Shape** | **Size**  **(mm)** | **Laminar size**  **(mm^2^)** | **Blade class** | **Petiole**  **(mm)** | **Primary veins (1°)** | **Secondary veins (2°)** | **Tertiary veins (3°)** |
| --- | --- | --- | --- | --- | --- | --- | --- | --- | --- | --- | --- |
| *Cercis canadensis* | acuminate | cordate | entire | broadly ovate heart-shaped | 70–127 x 50–100 | 3500–12700 | mesophyll | 38–64 | actinodromous 7–9 main veins | brochiododromous | alternate percurrent |
| *Tinospora cordifolia* | acuminate | cordate | entire | broadly ovate heart-shaped | 80–110 x 70–90 | 5600–9900 | mesophyll | 40–60 | actinodromous  5–7 main veins | brochiododromous | alternate percurrent |
| *Matelea carolinensis* | acuminate | cordate | entire | ovate heart-shaped | 50–100 x 20–50 | 1000–5000 | mesophyll | 30–60 | actinodromous 5 main veins | brochiododromous | opposite percurrent |
| *Catalpa bignonioides* | acuminate | cordate | entire | broadly ovate heart-shaped | 150–300 x 70–200 | 10500–60000 | macrophyll | 70–160 | actinodromous 5 main veins | brochiododromous | alternate percurrent |
| *Ipomoea purpurea* | acuminate | cordate | entire and slightly sinuate | cordate | 20–100 × 20–100 | 10000–10000 | mesophyll | 25–60 | actinodromous  3–5 main veins | brochiododromous | alternate percurrent |
| *Thespesia populnea* | acute to acuminate | cordate | entire | orbicular or ovate | 50–127 x 55–150 | 27500– 19050 | macrophyll | 50–100 | actinodromous  5–7 main veins | brochiododromous | alternate percurrent |
| cf. *Ipomoea* | acuminate | cordate | entire and slightly sinuate | cordate | 60–100 × 80–120 | 4800–12000 | mesophyll | 30 | actinodromous  5 main veins | brochiododromous | alternate percurrent |

**Supplementary Table S1.** Comparative morphological chart of extant angiosperm leaves related to cf. *Ipomea* fossil leaves.
