# Supplementary material for: Marginal leaf galls on Pliocene leaves from India indicate mutualistic behavior between Ipomoea plants and Eriophyidae mites
Source: Sci Rep. 2023 Apr 7;13:5702. doi: 10.1038/s41598-023-31393-2 (PMC10082081; doi:10.1038/s41598-023-31393-2)
Supplement: Supplementary file 10 — Supplementary Table S2. [file 41598_2023_31393_MOESM10_ESM.docx]

**Table S2: List of plant species with their specific galls**

| **Host plants** | **Causal organisms** | **Salient features of marginal galls** | **References** |
| --- | --- | --- | --- |
| *Tenninalia arjuna* (Combretaceae) | *Megatrioa hirsutam* (Homoptera) | Leaf-galls on margin, subspherical, sessile, smooth, brown, unilocular pouch-gall, with a hypophyllous crateriform depression, surrounded by a raised marginal rim and with an ostiole in the centre, leading into the gall cavity, size 2 mm in diameter | Mani, 1973 |
| *Madhuca longifolia*  (Sapotaceae) | Itonididae (Diptera) | Leaf galls hemispherical or strongly convex, rarely globose, rugose, brown, solid, fleshy, hard, indehiscent, persistent, sessile on leaf margin; sometimes 2 or 3 galls agglomerate in a linear row; the site of the galls having a conspicuous depression; gall cavity central, oval; size 1-5 mm in diameter | Mani, 1973 |
| *Avicennia officinalls* (Acanthaceae) | *Eriophyes* sp. (Acarina) | Leaf galls are sessile, sub-globose, agglomerate or solitary, indehiscent, solid, soft, fleshy, pouch-gall, with fine soft, fleshy, closely crowded, pale hair-like emergences on the outer surface; the gall tissue with irregular, erineum-filled, narrow and nearly obliterated interspaces; on the opposite side of the leaf blade, the site of the galls are indicated by a small discolored depression, in which lies the irregular ostiole. The galls arise in large numbers in a linear series on the leaf margin or along the midrib; size 2-4 mm in diameter. | Mani, 1973 |
| *Piper nigrun* (Piperaceae) | *Gynaikothrips karnyi* Bagnall (Thysanoptera) | Leaf margin ' stitch ' gall, not fully described. | Mani, 1973 |
| *Loranthus* sp. (Loranthaceae) | Unknown Psyllidae (Homoptera) | Leaf-galls ovoid, ellipsoid or sub-globose, unilocular, fleshy, brownish pouch-galls on leaf margins and visible on both sides of the blade, about 10-15 mm long and 5-8 mm thick. | Mani, 1973 |
| *Ficus drupacea* (Moraceae) | Orthoptera ( ?) | Leaf-galls procecidia, sub-globose or fusiform, solid, hard, largely hypophyllous, on leaf margin; the epiphyllous portion is flat-sausage-shaped, smaller, and thinner than the hypophyllous portion, which is sub-globose or hemispherical; the surface reddish-brown cracked and fissured and bark-like in general appearance and corky in nature. In section, the gall presents a thick outer corky layer, size 10-15 mm in diameter. | Mani, 1973 |
|  | *Pauropsylla globuli* (Homoptera) | Leaf-galls globose, coriaceous, unilocular, greenish or brownish, dehiscent pouch-gall, developing on both sides of the leaf blade, but relatively more numerous on the upper surface than on the lower and particularly abundant near the leaf margins, size 5-6 mm in diameter. | Mani, 1973 |
| *Caryocar brasiliense*  (Caryocaraceae) | *Eurytoma* sp. (Hymenoptera: Eurytomidae) | The *Eurytoma* sp. galls are spherical, yellowish, and unilocular with whitish trichomes. | Mani, 1973 |
| *Quercus* sp. | *Andrlcus pruinosus*, *A. utriculus, A. pilulus; Neuroterus bassettii* *N. cockerelli;* *Dryophanta pulchripennis* | Galls globose, densely pruinose, thin-walled, leaf or ament gall, diameter ~ 3 mm, sometimes banded by a minute ridge, green or purplish, pubescent | Felt, 1940 |

**Supplementary Table S2.** Modern margin leaf galls description and their host plants and causal organisms.
