# Supplementary material for: Marginal leaf galls on Pliocene leaves from India indicate mutualistic behavior between Ipomoea plants and Eriophyidae mites
Source: Sci Rep. 2023 Apr 7;13:5702. doi: 10.1038/s41598-023-31393-2 (PMC10082081; doi:10.1038/s41598-023-31393-2)
Supplement: Supplementary file 11 — Supplementary Information. [file 41598_2023_31393_MOESM11_ESM.doc]

DT413 (Galling)

Description: A linear chain of small rounded black dots (*i.e.,* galls) along the margin of the leaf limb. Each gall is in a small depression at the edge of the limb. The galls are sessile, solid, solitary, and isolated from each other. Leaf galls are 0.5 ‒ 1.5 mm in diameter and 1 ‒ 4 mm apart.

Holotype specimen: SKBUH/PPL/JH/324A (see Figure 2A)

Host plant: *Ipomoea* L. (Family Convolvulaceae)

Inferred herbivore: Eriophyidae

Modern ecological analogue: Some similar observations have been done on the following modern leaves *Terminalia arjuna* (Roxb.) Wight & Arn. (Combretaceae), *Madhuca longifolia* (J. Konlg) J.F. Macbr. (Sapotaceae), *Avicennia officinalis* L. (Avicenniaceae), *Piper nigrum* L. (Piperaceae), *Loranthus* Jacq. (Loranthaceae), *Ficus drupacea* Thunb. (Moraceae), *Schima wallichii* (DC.) Korth. (Theaceae), *Alstonia scholaris* (Apocynaceae), *Ipomoea staphylina* (Convolvulaceae) and *Caryocar brasiliense* Cambess. (Caryocaraceae)
